# Theoretical Analysis of Efficient Thermo-Optic Switching on Si_3_N_4_ Waveguide Platform Using SiOC-Based Plasmo-Photonics

**DOI:** 10.3390/nano15040296

**Published:** 2025-02-15

**Authors:** Dimitris V. Bellas, Eleftheria Lampadariou, George Dabos, Ioannis Vangelidis, Laurent Markey, Jean-Claude Weeber, Nikos Pleros, Elefterios Lidorikis

**Affiliations:** 1 Department of Informatics, Aristotle University of Thessaloniki, 54124 Thessaloniki, Greece; e.labadariou@uoi.gr (E.L.); dabosgeorge@gmail.com (G.D.); npleros@csd.auth.gr (N.P.); 2 Center for Interdisciplinary Research and Innovation (CIRI-AUTH), Balkan Center, 57001 Thessaloniki, Greece; 3 Department of Materials Science and Engineering, University of Ioannina, 45110 Ioannina, Greece; i.vangelidis@uoi.gr (I.V.); elidorik@uoi.gr (E.L.); 4 Laboratoire Interdisciplinaire Carnot de Bourgogne (LICB) UMR 6303, Université de Bourgogne, 9 av. A Savary, BP47870, 21078 Dijon, CEDEX, France; laurent.markey@u-bourgogne.fr (L.M.); jean-claude.weeber@u-bourgogne.fr (J.-C.W.)

**Keywords:** photonic integrated circuits, silicon nitride photonic platform, silicon oxycarbide, thermo-optic switcher, plasmonic–photonic interferometer, multimode interference

## Abstract

Photonic integrated circuits (PICs) are crucial for advanced applications in telecommunications, quantum computing, and biomedical fields. Silicon nitride (SiN)-based platforms are promising for PICs due to their transparency, low optical loss, and thermal stability. However, achieving efficient thermo-optic (TO) modulation on SiN remains challenging due to limited reconfigurability and high power requirements. This study aims to optimize TO phase shifters on SiN platforms to enhance power efficiency, reduce device footprint, and minimize insertion losses. We introduce a CMOS-compatible plasmo-photonic TO phase shifter using a SiOC material layer with a high TO coefficient combined with aluminum heaters on a SiN platform. We evaluate four interferometer architectures—symmetric and asymmetric Mach–Zehnder Interferometers (MZIs), an MZI with a ring resonator, and a single-arm design—through opto-thermal simulations to refine performance across power, losses, footprint, and switching speed metrics. The asymmetric MZI with ring resonator (A-MZI-RR) architecture demonstrated superior performance, with minimal power consumption (1.6 mW), low insertion loss (2.8 dB), and reduced length (14.4 μm), showing a favorable figure of merit compared to existing solutions. The optimized SiN-based TO switches show enhanced efficiency and compactness, supporting their potential for scalable, energy-efficient PICs suited to high-performance photonic applications.

## 1. Introduction

Photonic integrated circuits (PICs) have emerged as a transformative technology, providing a compact, efficient platform for manipulating and processing optical signals [1,2]. Their versatility enables applications across a wide array of fields, including data communications and telecommunications [3], biomedical diagnostics [4], quantum computing [5], positioning and navigation [6], spectroscopy [7], and atomic clocks [8]. By integrating multiple optical components, such as modulators, photodetectors, and lasers, PICs reduce system complexity, size, and power consumption, which is critical for scalable, high-performance photonic systems [9]. A critical capability within PIC technology is efficient light modulation and switching. Modulators and switches control optical signals by either modifying the refractive index (phase tuning) or adjusting optical losses (amplitude tuning) within the guiding material. These devices leverage various physical mechanisms—such as electro-optic (EO), thermo-optic (TO), phase transition (PT), and electrochemical metallization (ECM) effects—each optimized for specific operational requirements [10,11]. Key performance metrics, including switching speed, power consumption, and optical losses, determine the suitability of these devices for different applications. For instance, high-speed EO modulators are crucial in telecommunications for rapid data transmission [12,13], while TO switches, which offer advantages in simplicity, cost-effectiveness, and energy efficiency, are ideal for wavelength filtering, optical routing in data centers [14,15], and photonic neural networks (PNNs) as weighting elements in training and inference applications [10] where speed is less critical. Consequently, the choice of modulation technology is highly application-dependent, prioritizing performance for targeted operational criteria.

Traditionally, silicon-on-insulator (SOI) platforms have dominated the PIC landscape due to their compatibility with CMOS processes, allowing for mass production at relatively low costs [1,9]. However, SOI platforms face several challenges, particularly in terms of high optical losses, limited transparency, and thermal instability. These challenges have prompted exploration into alternative materials, with silicon nitride (SiN) emerging as a promising CMOS-compatible platform for low-loss, high-performance photonic applications. SiN offers excellent transparency across a broader wavelength range, making it well suited for applications demanding both high optical fidelity and thermal stability [16,17]. SiN photonics continue to gain traction across diverse fields, from biosensing to quantum computing, where requirements such as a wide transparency range (400 nm–2350 nm), low optical loss (<1 dB/m), moderate index contrast, and compatibility with wafer-scale manufacturing are critical [18,19,20,21,22,23]. Despite their unique advantages, SiN platforms face challenges in achieving reconfigurability comparable to SOI-based alternatives, particularly through the development of energy-efficient TO phase shifters. Current SiN-based TO phase shifters often contend with low thermo-optic coefficients (TOC), i.e., $dn/dT=2.45\times 10^{-5}\;RIU/K$*,* large millimeter-scale footprints [24], and high power dissipation [25], limiting their adoption in applications where energy-efficient TO functionality is essential.

To address these limitations, one promising approach enhances the TO effect in SiN platforms through silicon oxycarbide (SiOC) film deposition atop, leveraging its higher TOC $dn/dT=2.5\times 10^{-4}RIU/K$. This strategy involves precise waveguide (WG) engineering to increase modal confinement in regions with high TOC materials, amplifying TO efficiency [26]. While this optimization shows potential, practical deployment and robust performance evaluation are limited by the challenge of integrating metal heaters atop SiOC layers to actively control the switching. Alternative approaches, including hybrid plasmonic heaters integrated onto SiN and Si platforms, have also been investigated to improve TO tuning efficiency and enable ultra-compact devices, though often at the cost of increased propagation losses [27,28].

**Figure 1:**
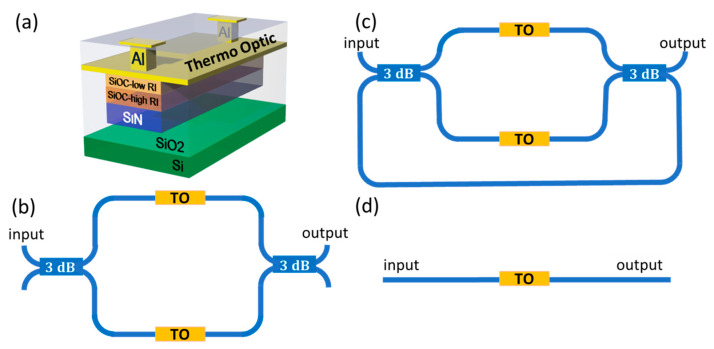
(**a**) Cross-sectional view of the active thermo-optic (TO) layered stack. Schematics of the thermo-optic interferometer architectures examined: (**b**) Mach–Zehnder Interferometer (MZI) applicable for both symmetric and asymmetric configurations, (**c**) asymmetric MZI with ring resonator-assisted configuration, (**d**) single-arm configuration.

In this work, we present an optimization process for TO switches, focusing on the configuration and material stack of the TO element to enhance power efficiency, reduce device length, and minimize insertion losses in SiN-based PICs. Specifically, we use a fully validated (against experimental results) simulation method [29,30], to explore and numerically optimize different interferometer architectures on a SiN photonic WG platform utilizing an Al/Low-Refractive-Index SiOC (LRI-SiOC)/High-Refractive-Index SiOC (HRI-SiOC) plasmonic WG as the TO switching element (Figure 1a). We exploit the high TOC of SiOC layers through the joule heating of Al contact to induce the appropriate phase difference between the interfered waves for efficient switching. We examine a double-arm MZI configuration where the switching is taking place through the single-mode photonic interference at the end of two branches at different configurations: (a) symmetric MZI (S-MZI) working at a full π applied external phase shift, (b) asymmetric MZI (A-MZI) working at π/2 applied external phase shift (Figure 1b), and (c) A-MZI ring resonator-assisted configuration (A-MZI RR) working at π/4 or π/6 applied external phase shift (Figure 1c). We also examine a single-arm (1-ARM) interferometer configuration where the switching is taking place in the output photonic WG, driven by the two plasmo-photonic modes of the TO WG (Figure 1d). We evaluate the different architectures in terms of losses, footprint, energy consumption, and switching time. High-performance interferometers feature low IL, large extinction ratio (ER), small footprint, high-frequency operation (fast switching time), and low power switching. Our optimal results compared to the state of the art show that our approach significantly advances the efficiency, scalability, and integration of SiN-based PICs compared to existing methods for TO switching, laying the groundwork for compact, energy-efficient, and scalable PICs tailored for a wide range of high-performance photonic applications.

The rest of the manuscript is organized as follows: in the next section, we present the state of the art and the opto-thermal methodology for the optimization and evaluation of TO switchers, developing a semi-analytical model (validated by 3D FDTD simulations) for the fast screening of the switcher parameters. Subsequently, we define an optimization process to study the functionality of the whole ΜΖΙ, specifying the optimal SiOC layers and the device geometry. Furthermore, we perform thermal simulations for the optimization of the power consumption and switching time and specify the device configuration for the optimal thermal dissipation and extract the power consumption of the device. In the Results and Discussion section, we utilize the developed opto-thermal models to optimize the performance of the different TO interferometer architectures, i.e., S-MZI, A-MZI, A-MZI-RR, and 1-ARM. In doing this, we extract the optimum performance metrics for each configuration, i.e., I.L., power consumption, and footprint. Finally, we conclude by comparing the performance metrics for the different examined architectures.

## 2. Opto-Thermal Optimization

### 2.1. Relation to State of the Art and Optimization Methodology

To benchmark our SiN-based TO phase shifter optimization results against the state of the art (SotA), we compare against two alternatives: (a) existing TO shifters on SiN platforms and (b) dielectric-loaded surface plasmon polariton TO phase shifters. Specifically, Table 1 highlights and compares the performance metrics of several relative experimental works. In terms of standard SiN PICs, these include the following: the first demonstration of TO switching employing a 2 × 2 Mach–Zehnder Interferometer (MZI) configuration, with SiN functioning as both the WG core and the TO material [24]; a 4 × 4 matrix switch enabling intrinsically polarization-insensitive operation [25]; MZI structures with compact thermal tuners as multi-heated folded optical WGs [31]; and a silicon-rich silicon nitride (SRN) WG platform taking advantage of the higher TOC of SRN $dn/dT=1.65\times 10^{-4}RIU/K$ [32]. Another approach in the literature noted in Table 1 involves plasmonic TO switches in a simplified design where the heating wire also serves as the plasmonic metal supporting surface plasmon polaritons (SPPs) and a high TOC dielectric layer near the metal to enhance performance in the so-called dielectric-loaded SPP (DLSPP) configuration [33,34,35,36,37,38]. Such DLSPP-based MZI switching devices had been demonstrated by employing the following: polymethylmethacrylate (PMMA) with $dn/dT=-1.05\times 10^{-4}\;RIU/K$ on MgF_2_ [33,34]; cycloaliphatic acrylate polymer (Cyclomer) with $dn/dT=-2.95\times 10^{-4}\;RIU/K$ and a low thermal conductivity Cytop (0.12 W/mK) buffer layer before the substrate [35]. Finally, full device demonstrations in Table 1 include the following: a PMMA-based asymmetric MZI (A-MZI) on SOI employing Si-based coupler stages and TO PMMA-based DLSPP WGs as active arms, employed in Wavelength Division Multiplexing (WDM) with error-free switching functionality at 4×10 Gb/s incoming data traffic [37]; a Cyclomer-based asymmetric MZI (A-MZI) WDM evaluated under 10 Gb/s data traffic [38]. It is important to note here, however, that DLSPP switches based on polymer TO active layers are not compatible with CMOS technology.

**Figure 2:**
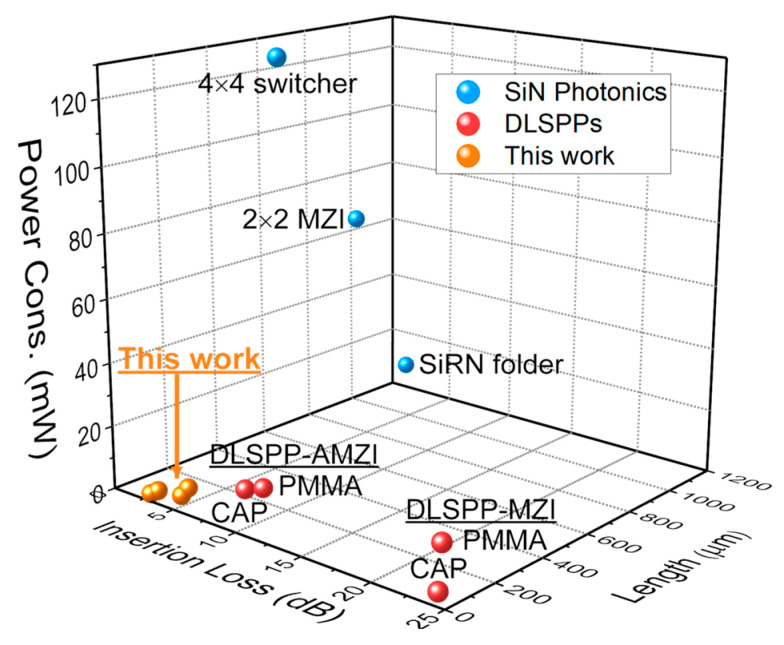
Specify our work within the state-of-the-art TO switches on SiN platforms and DLSPPs, as presented in Table 1.

To develop our optimization strategy, we note that the key performance metrics, i.e., integration density, energy efficiency, and signal quality, are adequately quantified by the three key parameters of device length L (μm), power consumption P (mW), and insertion losses IL (dB), respectively. In general, some trade-offs exist between them and are important when considering specific application requirements. For example, a smaller device footprint promotes faster heat spreading and thus may require higher power per device length to reach and maintain a certain switching temperature; a longer device length will introduce higher propagation (and thus insertion) losses; higher losses, on the other hand, may require more total operating power but have no impact on the TO switching power quoted here. Thus, we consider all three separately in this work. In principle, it is desirable for all three to be as small as possible. In Figure 2, we visually compare the optimized device metrics found in our study against the SotA (shown in Table 1) to find that our optimized devices define the smallest volume L·P·IL in the corresponding 3D metric space. Consequently, we will use this volume as a convenient additional figure of merit, i.e., FOM=L·P·IL [μm·mW·dB]. This FOM methodology was employed in previous works to compare the performance of modulator geometries across different technology platforms [39,40]. Note that while the inclusion of IL in dBs introduces a logarithmic element to the metric, it aligns well with standard photonic industry practices due to its ability to reflect the exponential nature of optical signal attenuation and/or cascaded systems where losses accumulate significantly [40]. This formulation ensures that the FOM remains practical, interpretable, and relevant to a wide range of photonic system applications. However, we also stress that it should be adapted when considering specific application requirements, e.g., by prioritizing power efficiency for data centers or footprint for dense photonic integration.

### 2.2. Optical Modeling of SiOC TO WGs

**Figure 3:**
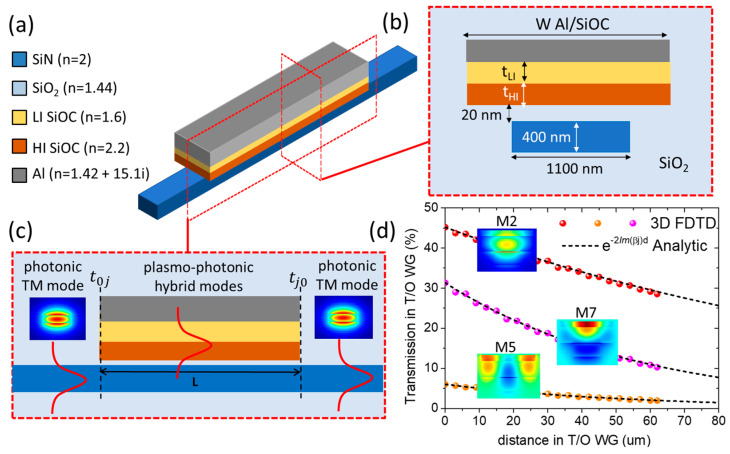
(**a**) A 3D schematic illustration of the TO active WG showing the stratified material stack and the corresponding refractive indices. (**b**) A 2D cross-section highlighting the dimensions considered for optimization, specifically the width of the TO WG ($W_{TO}$) and the thicknesses of the SiOC layers. (**c**) A 2D side view depicting the injected transverse magnetic (TM) photonic mode, which couples to various plasmo-photonic hybrid modes supported by the TO WG stack (see insets in (**d**)) and then decouples into the SiN photonic mode. (**d**) Three-dimensional FDTD simulations demonstrating the coupling transmission (at zero distance, i.e., the front interface) and propagation of the dominant plasmo-photonic modes (M2, M5, M7) as a function of distance in the TO waveguide stack. The dotted lines represent the corresponding Beer–Lambert law for each mode, showing perfect agreement with the 3D FDTD simulations. The cross-sectional parameters of TO WG are as follows: $W_{TO}=2.1\;\mu m$, $t_{LI}=180\;nm$, $t_{HI}=480\;nm,$ and L=62 μm.

Starting with the optical simulations, we define a semi-analytic model and an optimization process to maximize the transmission efficiency of the plasmonic SiOC-based TO WG, which is used as an active switching element in the ΜΖΙ architectures. In this theoretical model, we consider the Al/LRI-SiOC/HRI-SiOC/SiO_2_ (20 nm) above the SiN photonic WG embedded in SiO_2_ (see 3D and 2D schematics in Figure 3). We investigate this stratified TO switcher for TM operation at the telecom wavelength 1550 nm. The low-index SiOC is placed close to Al contact to reduce the propagation losses and the high-index below to increase the TO effect. The target cross-section for the SiN photonic WG is  100 nm×400 nm and the refractive index is n=2 [41], which supports single-mode operation for TM polarization (inset in Figure 3c). For the performance optimization of plasmonic SiOC TO WG, we explore a wide range of thicknesses for both low- and high-index SiOC layers ($t_{LI}$, $t_{HI}$) and different widths for the Al/SiOC stack ($W_{TO}$), as is depicted in Figure 3b. The refractive index of Al is $n_{Al}=1.42+i15.1$ [42]. The refractive indices of low- and high-index SiOC layers are as follows: $n_{SiOC}^{low}=1.6+i0.0001$ [43] and $n_{SiOC}^{high}=2.2+i0.0001$ [26], respectively. In optical calculations, we apply the TOC only in the SiOC layers, which is $dn/dT=2.5\times 10^{-4}\;RIU/K$ for high-index [26] and $1.0\times 10^{-4}\;RIU/K$ for low-index SiOC [43,44]. The TOC of the other dielectrics is quite low in the order of $10^{-5}\;RIU/K$ [26] and for simplicity not considered in our calculations.

Moving to the operation of the plasmonic SiOC-based TO WG, we found several plasmo-photonic hybrid modes supported by the stratified stack, i.e., M2, M5, and M7 (see insets in Figure 3d). Thus, the injected TM photonic mode couples to multiple plasmo-photonic modes supported by the Al/SiOC stack (Figure 3c). Each mode j propagates independently in the TO WG according to its propagation constant $\beta_{j}$, in excellent agreement with the exponential Beer–Lambert law as is depicted in Figure 3d for the three dominant modes. At the far end of the TO WG, the modes interfere back into the photonic (single) mode of the SiN WG. To facilitate structure optimization, we develop a semi-analytical model, similar to the model presented in our previous works [29,30], to resolve the multimode operation within the SiOC element. This results in total transmission including the interface and the propagation losses:

(1) $${T=\left|t\right|}^{2}={\left|\sum t_{0j}e^{i\beta_{j}L}t_{j0}\right|}^{2}$$

where $t_{0j}$ is the transmission amplitude of the SiN photonic mode (0) to the TO j plasmo-photonic mode at the front interface, $\beta_{j}$ is the propagation constant of the j plasmo-photonic mode, L is the length of the TO WG, and *t*_j0_ is the transmission amplitude of the TO j plasmo-photonic mode to the SiN photonic mode (0) at the back interface of the TO WG.

Assuming that there is no mode conversion in the plasmo-photonic TO WG and symmetry in the input/output coupling regions, we can set $\;t_{j0}=t_{0j}^{*}$, simplifying Equation (1) to the following:

(2) $$T={\left|\sum {\left|t_{0j}\right|}^{2}e^{i\beta_{j}L}\right|}^{2}$$

where ${\left|t_{0j}\right|}^{2}$ now represents the coupling coefficients from the input photonic mode to the *j* plasmo-photonic mode and back. The ${\left|t_{0j}\right|}^{2}$ parameters are extracted from the 2D mode calculations (Lumerical Waveguide Simulator [45]), along with the propagation constant $\beta_{j}$. This semi-analytical model was previously utilized in the design and optimization of a bimodal interferometric plasmo-photonic refractive index sensor, as detailed in our earlier studies [29,30], which guided the corresponding experiments. A comparison of the experimental results with the theoretical predictions revealed excellent agreement, demonstrating that the analytical model accurately captures the bimodal interferometric response.

**Figure 4:**
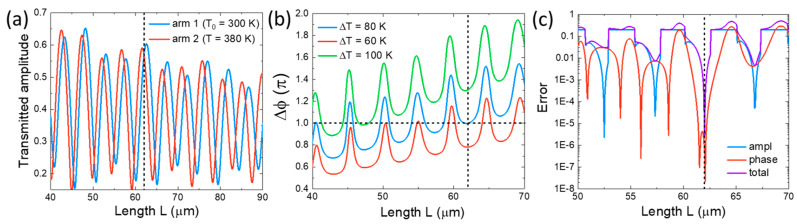
(**a**) Transmitted amplitude in each arm as a function of the TO WG length, showing two equal-amplitude points per period. (**b**) Phase difference between the two arms for three different temperature increments, plotted against the TO WG length. (**c**) Amplitude, phase, and total error function (i.e., divergence from the ideal π phase-shift operation) as a function of the TO WG length. Dashed lines in each graph indicate the optimal TO WG length (L=62 μm). The cross-sectional parameters of the TO WG are consistent with those shown in Figure 3.

Using the semi-analytical model, we develop an optimization procedure to analyze the functionality of the entire MZI. We focus on the case of an S-MZI, where the SiOC-based TO WGs in both arms are of equal length. To achieve perfect cancellation of the interfering photonic modes at the output, both arms must deliver identical amplitude and a precise π phase difference, accomplished by heating the SiOC WG in one arm. This optimization is complex due to the multimode operation of the TO waveguide. Specifically, the transmitted amplitude in each arm—where the first arm remains at room temperature ($T_{0}=300\;K$) and the second arm is heated to T=380 K—varies with length, displaying two equal-amplitude points per period (Figure 4a), with the lower point being avoided. Additionally, the phase difference between the two arms fluctuates with length, yielding multiple bias-dependent solutions for the targeted Δφ=π. By adjusting the temperature bias, we can fine-tune the TO waveguide length to achieve the desired phase shift (Figure 4b).

We define a mathematical error function that captures the requirements for both amplitude and phase (i.e., the sum of squared deviations from the target conditions): $E_{amp}={\left(\left|t_{2}\left(T\right)\right|-\left|t_{1}(T_{0})\right|\right)}^{2}$ and $E_{phase}={\left(\Delta \varphi -\pi \right)}^{2}/\pi^{2}$ where $\left|t_{1}(T_{0})\right|$ and $\left|t_{2}\left(T\right)\right|$ are the transmission amplitudes of the cold arm (arm 1) and the heated arm (arm 2), respectively, and $\Delta \varphi =\varphi_{2}(T)-\varphi_{1}(T_{0})$ is the phase difference between the two arms. Then, the total error function is as follows: $E_{total}=E_{amp}+E_{phase}$, which is used in a numerical optimization process to pick the optimal device parameters. An example of this process is shown in (Figure 4c). The optimized device achieves highly effective interference, ensuring a high ER by default, so ER is not used as an evaluation metric in our interferometric configurations, which is defined as follows:

(3) $$ER=10\cdot log\left(\frac{T_{max}}{T_{min}}\right)$$

where $T_{max}$ is the maximum total transmission of the MZI, given by the interference of two photonic modes at the end of MZI with TO WGs in both arms at room temperature:

(4) $$T_{max}=\frac{1}{4}{\left|t_{1}\left(T_{0}\right)+t_{2}(T_{0})\right|}^{2}$$

and $T_{min}$ is the minimum total transmission of the MZI, given by heating the TO WG at the second arm:

(5) $$T_{min}=\frac{1}{4}{\left|t_{1}\left(T_{0}\right)+t_{2}(T)\right|}^{2}\;$$

Here, the factor of 1/4 serves as an energy normalization factor, accounting for the fact that the power of the incident photonic mode is split in half as it enters the two MZI branches. Each transmission amplitude is derived from a mode solver for a single-incident photonic mode.

**Figure 5:**
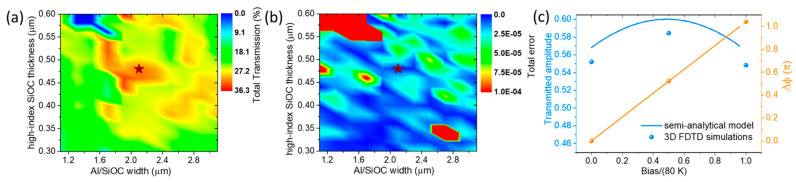
(**a**) Total transmission ($T_{max})$ of S-MZI as a function of high-index SiOC thickness and Al/SiOC width for 180 nm low-index SiOC and ΔT=80 K. (**b**) The corresponding total error function, with a star point indicating a high-transmission region with minimal total errors. (**c**) Full 3D FDTD simulations validating the semi-analytical model in both amplitude and phase, as a function of normalized bias (the ratio of the applied temperature difference to ΔT=80 K), for the optimum case indicated by the star point in (**a**,**b**).

We subsequently demonstrate the developed optimization process by investigating the geometric parameters of the TO WG. Specifically, we explore a wide range of Al/SiOC widths as a function of high-index SiOC thickness, with the low-index SiOC thickness fixed at 180 nm. Figure 5 presents a color map of the total transmission for the S-MZI (Figure 5a) and the corresponding total error function (Figure 5b) as a function of these two free parameters. A region of high transmission and minimal error with good tolerance in terms of thickness and width is identified. The star point indicates the optimal balance between maximum transmission and error, defined at a high-index SiOC thickness of 480 nm and an Al/SiOC width of 2.1 μm. This configuration achieves 32% maximum total transmission ($T_{max}$), corresponding to 4.9 dB total IL (i.e., $IL\left(dB\right)=-10\cdot {log}_{10}\left(T_{max}\right)$), encompassing coupling-in, propagation, and coupling-out losses with a TO WG length of $L_{1}=L_{2}=62\;\mu m$.

For this optimal configuration, we validate the semi-analytical model using full 3D Finite-Difference Time-Domain (FDTD) simulations. These simulations are conducted using Lumerical’s 3D FDTD Electromagnetic Simulator [45], modeling the Al/LRI-SiOC/HRI-SiOC/SiO_2_ (20 nm) stack above the SiN photonic WG. The SiOC thermal response is evaluated for three temperature differences: 0 K, 40 K, and 80 K. From these simulations, we extract the amplitudes and phases of the waves coupled back into the SiN photonic mode after passing through the Al/SiOC stack, accounting for total IL, including front interface coupling (SiN WG to SiOC TO WG), propagation losses in the SiOC TO WG, and back interface coupling (SiOC TO WG to SiN WG). Comparing the semi-analytical model with the exact 3D FDTD results (Figure 5c) shows excellent agreement for both the amplitude and phase across the applied temperature biases, fully validating our model. Notably, the requirement for equal amplitudes at 0 K and 80 K bias to achieve good interference is accurately reproduced by the 3D FDTD simulations. Therefore, the developed semi-analytical model serves as a reliable and efficient optimization tool (requiring only 2D cross-sectional simulations) to design experiments for SiOC-based TO WGs or similar devices, i.e., TiO_2_-based TO WGs [46].

### 2.3. Thermal Modeling of SiOC TO WGs

**Figure 6:**
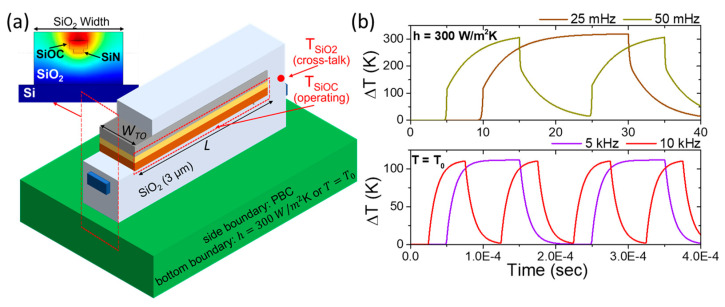
(**a**) Schematic illustration of the thermal model, showing the configuration of materials, temperature monitoring points, and the oxide layer width used for thermal isolation. The inset provides a cross-sectional view of the thermal simulations. (**b**) Transient temperature rise in the SiOC layer under varying operating frequencies and thermal dissipation conditions, including a heat exchange coefficient ($h=300\;W/m^{2}$) and a fixed room temperature ($T=T_{0}$). These simulations are conducted for the isolated SiO_2_ configuration shown in (**a**), where $W_{TO}=2.1\;\mu m$ and L=100 μm.

We now proceed to thermal simulations aimed at optimizing the switching time and power consumption. Specifically, to investigate the switching time, we analyze the effect of thermal dissipation in the device, considering two scenarios: a back-side convection with a given heat transfer coefficient and back-side active cooling fixed at room temperature. For energy consumption analysis, we explore two geometric thermal management configurations: one where the device is fully encapsulated in SiO_2_ and another where SiO_2_ is selectively etched away to match the SiOC width, as illustrated in the schematic of Figure 6a. Additionally, we developed a thermal model to evaluate the device’s power consumption. The heat transfer modeling was conducted using the 3D Finite Element Method (FEM) in the COMSOL simulator [47], with the thermal properties of each material detailed in Table 2. The layer sequence and thicknesses from top to bottom are as follows: SiO_2_ (1 μm)/SiOC (0.8 μm)/SiN (0.4 μm)/SiO_2_ (3 μm)/Si (675 μm). The computational cell is set to 400 × 400 μm^2^, with Periodic Boundary Conditions (PBCs) applied at the sides. We applied a time-dependent heat flux with a peak power of 10 mW on the top surface of the SiOC layer, simulating the power provided by joule heating from the Al contact. This setup allowed us to monitor the heating and cooling response of the device. Specifically, we tracked the average temperature increase within the SiOC volume to determine the operating temperature and observed the point temperature rise on the SiO_2_ side to assess thermal cross-talk between neighboring TO devices.

In Figure 6b, we analyze the impact of heat dissipation on the switching time of the SiOC TO WG. To perform this, we apply various thermal dissipation schemes at the bottom boundary of the device and assess the temperature response under different frequencies of the applied thermal load. Firstly, we consider a silicon back-side convection scenario with a heat transfer coefficient of $h=300\;W/m^{2}K$. We observe adequate switching performance from the hot to cold states within the low-frequency range of 25 mHz to 50 mHz (as shown in the upper graph of Figure 6b). However, this dissipation method results in slow thermal equilibrium with the silicon back-side, making it unsuitable for efficient TO switching. Next, we evaluate a bottom boundary condition maintained at room temperature ($T=T_{0}$) assuming an active thermoelectric cooler (TEC) at the silicon back-side. Under this configuration, we achieve exceptional switching performance at 10 kHz, indicating rapid operation for TO switching (see the lower graph in Figure 6b). This operational frequency aligns with previous theoretical studies on DLSPP-based TO shifters [36] and TO shifters on a SiN platform [51]. Thus, using a TEC for the back-side thermal dissipation proves to be an effective solution, enabling faster thermal stabilization and improved switching efficiency.

When comparing the switching times of the two thermal dissipation schemes, we observe that the switching time is more than 5 orders of magnitude shorter when using a bottom boundary fixed at $T=T_{0}$. To better understand this significant difference, we qualitatively examine the system’s energy conservation. The energy added to the system is determined by the input power ($P_{in}$) multiplied by the time (t) required for the system to dissipate this energy (i.e., the cooling time). This relationship can be expressed as follows: $VC\Delta T_{sub}=P_{in}t$ where V is the volume of the system, C is the effective volumetric capacity of the system, and $\Delta T_{sub}$ is the temperature rise in the substrate. Additionally, the input power dissipates from the system according to the heat transfer coefficient (h) as follows: $P_{in}=hA_{cell}\Delta T_{sub}$ with $A_{cell}$ the area of the computational cell. By combining these two equations, we derive the cooling time (t) as follows: $t=(VC)/(hA_{cell})$. This equation shows that the cooling time is inversely proportional to the heat transfer coefficient. Therefore, a bottom boundary maintained at $T=T_{0}$ (which corresponds to a very large h) results in a much faster cooling time. In contrast, a finite heat transfer coefficient of $h=300\;W/m^{2}K$ leads to a slower cooling process. This qualitative analysis aligns well with our findings from the 3D-FEM simulations, explaining the substantial difference in switching times between the two dissipation schemes.

**Figure 7:**
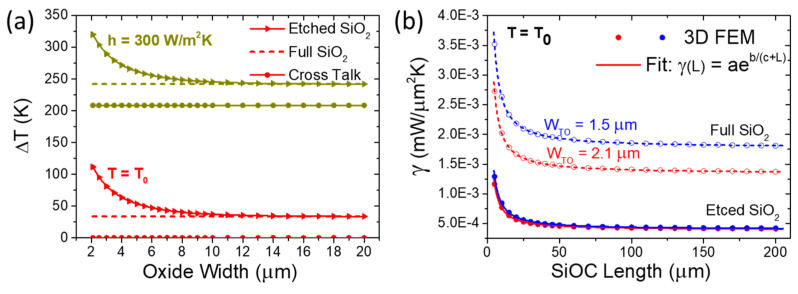
(**a**) The steady-state temperature rise, including both operating temperature and cross-talk effects, is analyzed as a function of the SiO_2_ width for two back-side thermal dissipation schemes: one with a heat exchange coefficient of $h=300\;W/m^{2}$ and the other with a fixed room temperature ($T=T_{0}$). For comparison, the corresponding results for a fully encapsulated SiO_2_ case are shown with dashed lines. The width and the length of TO WG are $W_{TO}=2.1\mu m$ and L=100μm, respectively. (**b**) The heat transfer coefficient of the TO WG is evaluated as a function of its length for two different TO WG widths ($W_{TO}=2.1\mu m$ and $W_{TO}=1.5\mu m$) for both etched and full top SiO_2_ assuming a Si back-side boundary condition fixed at room temperature. An exponential fit is applied to the simulated values to define the thermal model for the power consumption extraction of the SiOC TO WG.

To optimize the thermal performance of the TO device, we further investigate the impact of SiO_2_ width on both the operating and cross-talk temperatures under two different thermal dissipation schemes. As shown in Figure 7a, we explore a range of SiO_2_ widths from 2.1 μm (representing the isolated case where SiO_2_ is etched to match the SiOC width) up to 20 μm and compare these with a fully encapsulated SiO_2_ configuration. As expected, the SiOC volume exhibits a higher temperature rise for the back-side convection case ($h=300\;W/m^{2}K$) compared to the bottom boundary condition fixed at room temperature ($T=T_{0}$). Both thermal dissipation schemes demonstrate a universal trend, where the SiOC operating temperature significantly decreases with the increasing SiO_2_ width, eventually reaching the same temperature as the fully encapsulated SiO_2_ when the oxide width exceeds 10 μm. This highlights a crucial experimental limitation for thermal isolation, suggesting that reduced SiO_2_ widths can lead to significant power savings. Analyzing the cross-talk temperature, a substantial difference is observed between the two dissipation schemes. Specifically, for the back-side convection case, the cross-talk temperature ranges between 65% and 86% of the SiOC operating temperature, depending on the SiO_2_ width. This cross-talk temperature corresponds to a total substrate temperature rise of $\Delta T_{sub}=j/h=208\;K$, where $j=62.5\;kW/m^{2}$ represents the applied power flux. Such high cross-talk hinders the independent thermal control of individual devices in PICs, rendering this dissipation approach unsuitable despite its higher temperature rise. Conversely, the bottom boundary condition fixed at $T=T_{0}$ shows negligible cross-talk (~0.1%), making it a preferred solution for efficient thermal dissipation. Additionally, comparing the two SiO_2_ configurations, the isolated SiO_2_ setup results in a temperature rise more than three times higher than that of the fully encapsulated SiO_2_, due to reduced thermal contact with the substrate. This translates to over three times lower power consumption for the isolated SiO_2_ configuration for achieving a specific temperature increase.

Next, we establish a thermal model to estimate the power consumption of the TO element for various switcher lengths. We specifically focus on the thermal dissipation scenario where the Si back-side is maintained at a fixed room temperature, enabling high operating frequencies and minimal thermal cross-talk. Additionally, we investigate both full and etched SiO_2_ configurations for two WG widths: (a) 2.1 μm, optimized for the two-arm S-MZI and (b) 1.5 μm, optimized for the single-arm interferometer configuration (as defined later in the device optimizations). To achieve this, we use 3D FEM simulations to extract the heat transfer coefficient $\gamma (mW/{\mu m}^{2}K)$ of the SiOC TO element across a wide range of switch lengths, from 5 μm to 200 μm (see Figure 7b). The heat transfer coefficient reflects the thermal dissipation of the input heat flux, $q(mW/{\mu m}^{2})$, into the device’s surroundings, defined as γ=q/ΔT, where q=P/A. Here, P represents the input power of 10 mW applied to the SiOC area $A=W_{TO}L$, and ΔT denotes the average temperature rise within the SiOC volume.

To determine the power consumption of the TO WG for different lengths, we perform an exponential fit to the heat transfer coefficient values (derived from 3D-FEM simulations) as a function of length, expressed as $\gamma (L)=ae^{b/(c+L)}$, where *a*, *b*, and *c* are fitting parameters provided in Table 3. This formula provides an excellent fit with the 3D-FEM simulation data (see Figure 7b). Considering the physical implications of the fitted parameters, we examine two extreme cases: (a) as L→∞, γ converges to a constant value α, indicating a uniform spatial distribution of thermal flux (i.e., 1D distribution) determined by the physical properties of the system; (b) as L→0, γ approaches $\alpha e^{b/c}$, representing a radial (3D) spatial distribution of thermal flux with a higher transfer coefficient, also influenced by the system’s physical properties but in a different manner. In conclusion, this model enables us to calculate the power consumption for each WG length given a specific target temperature rise, using the formula: $P(W_{TO},L)=A\gamma \left(L\right)\Delta T$. We will utilize this model to evaluate the power consumption of TO WGs across different widths and thermal configurations.

In Figure 7b, we observe higher thermal transfer coefficients for the full SiO_2_ configuration compared to the etched SiO_2_ configuration for both TO WG widths, due to the increased thermal contact with the silicon substrate. For instance, for a TO WG with $W_{TO\;}=2.1\;\mu m$, L=62 μm, and ΔT=80K, the power consumption is 4.6 mW for the etched SiO_2_ configuration, compared to 15 mW for the full SiO_2_ configuration. This highlights the importance of thermal isolation, which can reduce energy consumption by more than three times. This result is consistent with previous theoretical studies on DLSPP-based TO shifters [36] as well as TO shifters on the SiN platform [51], where the implementation of isolation trenches along the heated sections of the WG significantly reduces power consumption.

Furthermore, the thermal transfer coefficient of the TO WG varies with width depending on the thermal configuration. In the full SiO_2_ case, we observe a higher coefficient for $W_{TO\;}=1.5\;\mu m$ compared to $W_{TO\;}=2.1\;\mu m$. This is due to the faster thermal dissipation in narrower TO WGs, which requires more power to achieve the same temperature increase. However, the narrower TO WG also has a lower effective volumetric capacity, leading to a higher temperature rise for the same thermal load. This latter effect slightly outweighs the former, resulting in lower power consumption for the narrower TO WG to achieve the same temperature increase as the wider one. In contrast, for the etched SiO_2_ configuration, a similar trend is observed, but with less intensity due to the reduced thermal contact with the silicon substrate. Hereafter, we will use the thermally isolated configuration for all power consumption calculations, assuming a temperature rise of ΔT=80 K and a fixed room temperature at the Si back-side, as this setup was found to support high operating frequencies and minimal thermal cross-talk.

## 3. Results and Discussion

### 3.1. Symmetric MZI

**Figure 8:**
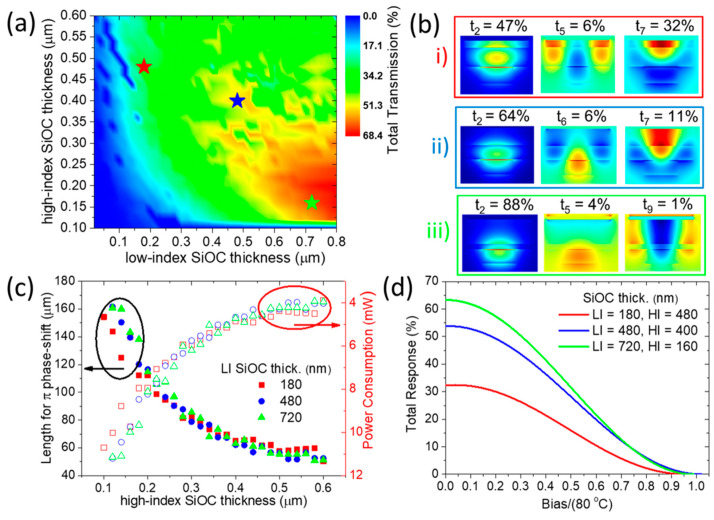
(**a**) Total transmission ($T_{max})$ of the S-MZI ($L_{1}=L_{2}$) as a function of high-index and low-index SiOC thickness for the optimized $W_{TO}=2.1\;\mu m$. (**b**) Spatial distribution of the electric field (Re(Ey)) for the plasmo-photonic modes supported by the TO WG, along with the coupling transmission from the SiN photonic mode to each plasmo-photonic mode, for the cross-sections marked by star points in (**a**). (**c**) Optimized WG length required for achieving a π phase shift and the corresponding power consumption as a function of high-index SiOC thickness, evaluated for three fixed low-index SiOC thicknesses. (**d**) Total modulation response as a function of normalized bias voltage for the cases indicated by the star points in (**a**).

To optimize the performance of different TO-based interferometer architectures, we apply the previously developed opto-thermal modeling framework. Starting with the S-MZI configuration (where $\;L_{1}=L_{2}$), maximum transmittance is achieved when there is zero phase difference between the two arms, meaning both arms are at the same temperature (e.g., $T_{0}=300\;K$), as is indicated by the Equation (4). Conversely, minimum transmittance occurs at a π phase difference, which can be induced by heating one arm (e.g., arm 2) to T=380 K, as is indicated by the Equation (5). To enhance the performance of the S-MZI, we investigate the impact of varying the thickness of both the high-index and low-index SiOC layers over a broad range (as shown in Figure 8a) while keeping the optimized WG width at $W_{TO\;}=2.1\;\mu m$. Interestingly, the results reveal a clear trend toward achieving a very high $T_{max}$ by reducing the high-index SiOC layer thickness while increasing the low-index SiOC layer thickness. To gain insight into the physical origin of this trend, we plot the spatial distribution of the electric field (Re(Ey)) for the fundamental and the two dominant plasmo-photonic modes supported by the TO waveguide (Figure 8b). Additionally, we evaluate the coupling transmission between the SiN photonic mode and each of these TO WG plasmo-photonic modes. For this analysis, we focus on three representative cross-sectional configurations that demonstrate high $T_{max}$ and minimal error, with good tolerance to variations in SiOC thicknesses, as indicated by the star points in Figure 8a.

In Figure 8a and b, we analyze the impact of varying SiOC layer thicknesses on the performance of the S-MZI. Starting with an optimized configuration at $t_{LI}=180\;nm$, $t_{HI}=480\;nm$, and $L_{\pi }=62\;\mu m$, we achieve a $T_{max}=32\%$, corresponding to 4.9 dB total IL (shown in Figure 8b(i)). By adjusting the layer thicknesses to $t_{LI}=480\;nm$, $t_{HI}=400\;nm$, and $L_{\pi }=62.2\;\mu m$, we improve the transmission to ${\;T}_{max}=54\%$ (2.7 dB IL) with a similar phase-shift length, as seen in Figure 8b(ii). Further optimization with $t_{LI}=720\;nm$, $t_{HI}=160\;nm$ yields a higher ${\;T}_{max}=63\%$ (2 dB IL), though with a longer phase-shift length of $L_{\pi }=143.27\;\mu m$ (Figure 8b(iii)). This trend toward a higher $T_{max}$ can be attributed to the spatial distribution of the electric field. For thinner low-index SiOC layers, the electric field is strongly confined to the metal surface, resulting in a significant portion of the coupling transmission being distributed among three plasmo-photonic modes. We categorize this behavior as the “strongly plasmonic” regime, characterized by elevated propagation losses due to metallic ohmic losses. As the low-index SiOC thickness increases and the high-index SiOC thickness decreases, the fundamental mode gradually transitions from “strongly plasmonic” to “strongly photonic”. In this regime, the electric field decouples from the metal surface and concentrates within the high-index SiOC and SiN waveguides. Consequently, propagation losses decrease while coupling transmission to the fundamental “strongly photonic” mode significantly improves due to its substantial spatial overlap with the incident SiN photonic mode. Additionally, the coupling to other plasmo-photonic modes is minimized, enabling efficient single-mode operation. Thus, by carefully tuning the low-index and high-index SiOC layer thicknesses, it is possible to shift from a “strongly plasmonic” mode (Figure 8b(i)) to a “strongly photonic” mode (Figure 8b(iii)), thereby optimizing the propagation losses and enhancing the overall device transmission efficiency.

We further analyze the optimized length ($L_{\pi }$) of the TO WG and its associated power consumption as a function of the high-index SiOC thickness, considering three fixed low-index SiOC thicknesses (see Figure 8c). Our findings indicate that the required length for achieving a π phase shift is significantly influenced by the high-index SiOC thickness, due to its approximately 2.5 times higher TOC compared to the low-index SiOC layer. Specifically, increasing the thickness of the high-index SiOC layer enhances the TO effect, resulting in a shorter $L_{\pi }$ and reduced power consumption. This configuration corresponds to a “strong plasmonic” operation mode, characterized by high TO sensitivity but at the expense of high insertion losses. Conversely, when the high-index SiOC layer is thin, the TO effect is diminished, leading to a longer $L_{\pi }$ and higher power consumption, which aligns with a “strong photonic” operation mode with low insertion losses. Also, we demonstrate for the three cases the overall response of the TO switcher as a function of the applied bias showing the maximum transmittance at 0 K bias and the minimum transmittance at 80 K bias (Figure 8d). To conclude, by tuning the thicknesses of the low- and high-index SiOC layers, we can move from “strong plasmonic” to “strong photonic” operation; each case presents specific advantages and disadvantages, as summarized in Table 4. A clear compromise emerges between consumption and insertion losses.

### 3.2. Asymmetric MZI

**Figure 9:**
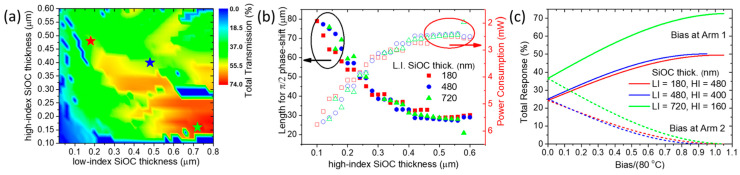
(**a**) Total transmission ($T_{max})$ of A-MZI ($L_{2}>L_{1}$) as a function of high-index and low-index SiOC thickness for optimized $W_{TO}=2.1\;\mu m$. (**b**) The optimized length ($L_{1}$) for π/2 phase shift and the corresponding power consumption as a function of high-index SiOC thickness, shown for the three fixed low-index SiOC thickness values. (**c**) Overall response of the switcher as a function of normalized bias for the corresponding cases indicated by the star points in (**a**).

We now proceed with the optimization of the A-MZI configuration. In this architecture, we aim for a π/2 TO phase shift, which requires only half the TO WG length ($L_{\pi /2}$) compared to the S-MZI. This optimization significantly increases the $T_{max}$ while reducing the energy required for switching. The asymmetry in the MZI is introduced by adjusting the lengths of the two TO WGs in the interferometer branches (e.g., $L_{2}>L_{1}$), thereby creating an inherent π/2 phase difference between the two arms. This design enables full tunability between 0 and π phase shifts by applying only an external π/2 phase shift, instead of a full π shift. This can be achieved either by driving the shorter arm ($L_{1}$) to achieve a 0 total phase difference and maximum MZI transmittance or driving the already π/2-biased longer arm ($L_{2}$) to achieve a π phase difference and minimum MZI transmittance. This approach allows for more efficient control of the MZI’s output with reduced power consumption, as demonstrated in the implementation shown in Figure 9c.

We applied the same optimization procedure used for the S-MZI configuration to the A-MZI and plotted the $T_{max}$ as a function of both low-index and high-index SiOC thicknesses, optimized for $W_{TO\;}=2.1\;\mu m$ (Figure 9a). Similar to the S-MZI case, increasing the low-index SiOC thickness while decreasing the high-index SiOC thickness allows a transition from “strong plasmonic” to “strong photonic” operation. The “strong photonic” mode provides better coupling transmission but requires a longer device length, leading to higher power consumption (Figure 9b). When comparing the performance of the S-MZI and A-MZI configurations, the A-MZI demonstrates superior efficiency due to its reduced device length, requiring only half the length for full modulation. Specifically, for the “strong plasmonic” case ($t_{LI}=180\;nm$, $t_{HI}=480\;nm$), the device length decreases from $L_{\pi }=62\;\mu m$ to $L_{\pi /2}=28.51\;\mu m$ ($L_{2}=28.72\;\mu m$), resulting in a reduction in energy consumption per switching event from 4.65 mW to 2.46 mW and IL from 4.9 dB to 3 dB. On the other side for the “strong photonic” case ($t_{LI}=720\;nm$, $t_{HI}=160\;nm$), the device length reduces from $L_{\pi }=143.27\;\mu m$ to $L_{\pi /2}=64.46\;\mu m$ ($L_{2}=64.69\;\mu m$), with energy consumption decreasing from 10 mW to 4.8 mW and IL from 2 dB to 1.4 dB.

The transition from S-MZI to A-MZI architecture shows a more significant reduction in insertion losses for the “strong plasmonic” case compared to the “strong photonic” case. This is due to the higher propagation losses in the plasmonic mode, despite a greater reduction in device length in the photonic mode. Additionally, the overall performance of the A-MZI modulator as a function of applied bias is illustrated, showing maximum transmittance at 0 K bias and minimum transmittance at 80 K bias (Figure 9c). In summary, these optimization maps serve as guidelines for the fabrication of efficient TO switchers in both S-MZI and A-MZI configurations, providing valuable insights into the trade-offs between power consumption and transmission efficiency.

### 3.3. Asymetric-MZI Ring Resonator-Assisted

**Figure 10:**
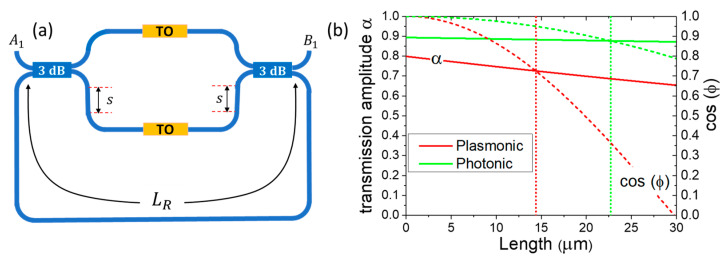
(**a**) Schematic of A-MZI ring resonator (RR)-assisted configuration. (**b**) The transmission amplitude and the cosine of the acquired phase φ upon heating one arm at ΔT=80 K. The vertical lines indicate the lengths corresponding to the critical coupling condition, where a=cos⁡(φ) for both “strong plasmonic” and “strong photonic” operational modes.

To further reduce the TO WG length and power consumption per switching of the device, we explore the A-MZI ring resonator (RR)-assisted configuration. This involves introducing a feedback loop ($L_{R}$) in the MZI, where the output from the bottom right port is fed back to the bottom left port, as shown in Figure 10a. Using a Transfer Matrix Model (TMM) that takes into account the coupling matrices of the 3 dB splitters and the propagation matrix of the MZI, along with the feedback ring loop, we derive the normalized transmission of the A-MZI-RR configuration [52]:

(6) $$T={{\left|\frac{B_{1}}{A_{1}}\right|}^{2}=\left(\frac{a}{a_{R}}\right)}^{2}{\left|\frac{ae^{i\Phi_{T}}-cos\varphi }{1-acos\varphi e^{i\Phi_{T}}}\right|}^{2}$$

Here, $a=a_{C}^{2}a_{MZ}a_{R}$ represents the total transmission amplitude through the TO MZI configuration, where $a_{c}^{2}$ accounts for the transmission amplitude through the 3 dB splitter, $a_{MZ}$ for the transmission amplitude through the MZI, and $a_{R}$ for the transmission amplitude through the ring. $\Phi_{Τ}$ is the total phase introduced by a complete path (both MZI and ring), and φ is the phase shift acquired by each MZI arm due to the SiOC-based TO WG, with Δφ=2φ being the total phase difference between the MZI arms.

To achieve a specific target value of φ (e.g., for critical coupling as described below), we introduce an asymmetry between the MZI arms by adding an extra length to the bottom SiN WG arm, which corresponds to a phase shift φ. This extra length s is given by s=φ/2β, where β is the propagation constant of the SiN WG photonic mode. In this way, by applying a bias to the bottom TO WG, we achieve a phase difference of Δφ=2φ. Conversely, applying bias to the top arm results in Δφ=0.

The condition for the target value of φ is determined by noting that the minimum transmission occurs under the critical coupling condition $\Phi_{T}=2m\pi$ with m=1,2,3… At this point, the condition cosφ=a must be satisfied. At the specific wavelength where $\Phi_{T}=2m\pi$, maximum transmission is achieved by biasing the top TO WG arm such that the phase shift φ induced by the bottom MZI arm is counterbalanced, resulting in Δφ=0. This occurs when cosφ=1, giving the following:

(7) $$T_{\varphi =0}=\frac{a^{2}}{a_{R}^{2}}\;$$

We now explore the critical coupling condition of the A-MZI-RR configuration, assuming both TO WGs function as switching elements within the MZI for the previously defined “strong plasmonic” and “strong photonic” cases. For this analysis, we assume that all losses originate solely from the TO WG of MZI, with the rest of the components considered lossless, i.e., $a_{C}^{2}=a_{R}=1$. The transmission amplitude scales according to the relation:

(8) $$\left|t\right|=a=a_{0}e^{-\gamma L}$$

where $a_{0}\;$ represents the transmission amplitude through the front and back interfaces of the TO WG, and $\gamma \;({\mu m}^{-1})$ is a fitting parameter. These values in Figure 10b are determined to be $a_{0}^{plasmonic}=0.8$ and $a_{0}^{photonic}=0.9$ at L=0, corresponding to ~2 dB and ~1 dB interface losses for the “strong plasmonic” and “strong photonic” cases, respectively. The fitting parameters are found to be $\gamma_{plasmonic}=0.0067\;\mu m^{-1}$ and $\gamma_{photonic}=0.00084\;\mu m^{-1}$. The phase shift acquired upon heating one arm is expressed as follows:

(9) φ=fLΔT

where $f_{plasmonic}=0.00021\;\mu m^{-1}K^{-1}$ and $f_{photonic}=0.000088\;\mu m^{-1}K^{-1}$(with φ in π units). These findings are illustrated in Figure 10b, where we examine the critical coupling condition for each case, assuming ΔT=80 K.

For the “strong plasmonic” configuration, the critical coupling condition is achieved at L=14.4 μm, resulting in an estimated total loss of ~2.8 dB and a required phase shift of φ=π/4. In contrast, for the “strong photonic” case, critical coupling occurs at L=22.7 μm, with an estimated total loss of ~1.1 dB and a required phase shift of φ = π/6. In conclusion, transitioning from the A-MZI to the A-MZI-RR configuration further reduces the power consumption per switching event from 2.46 mW to 1.6 mW for “strong plasmonic” operation and from 4.8 mW to 2.1 mW for “strong photonic” operation.

### 3.4. Single-Arm Interferometer

**Figure 11:**
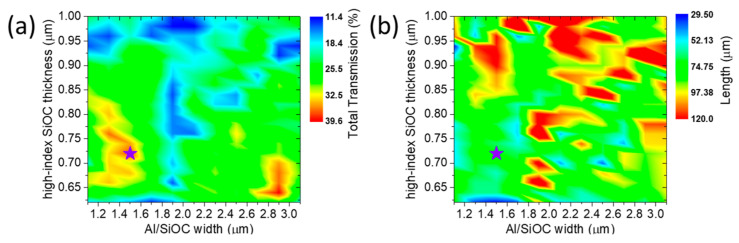
(**a**) Total transmission ($T_{max})$ of the 1-ARM interferometer as a function of high-index SiOC thickness and Al/SiOC width for a fixed low-index SiOC thickness of 180 nm. (**b**) The optimized length of the TO WG for the 1-ARM operation, corresponding to the configurations shown in (**a**). The star point in each graph denotes the optimal cross-section.

**Figure 12:**
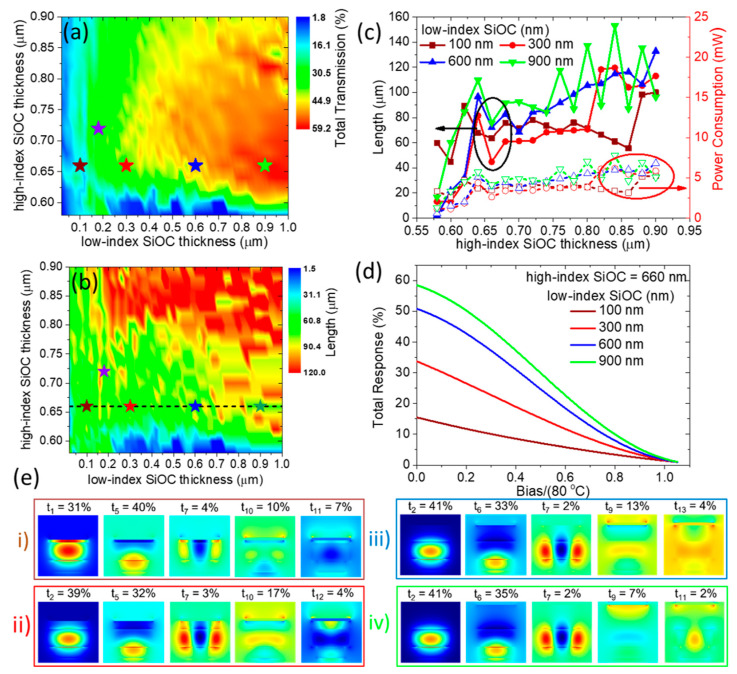
(**a**) Total transmission ${(T}_{max})$ of the 1-ARM interferometer as a function of high-index and low-index SiOC thickness for the optimized $W_{TO}=1.5\;\mu m$. (**b**) The optimized TO WG length for the 1-ARM operation depicted in (**a**). (**c**) The optimized TO WG length and corresponding power consumption as a function of high-index SiOC thickness for four fixed low-index SiOC thicknesses. (**d**) Total response of the switcher as a function of normalized bias for the respective cross-sections indicated by the star points in (**a**,**b**). (**e**) The real part of the electric field (Re(Ey)) spatial distribution of plasmo-photonic modes supported by the TO WG, along with the coupling efficiency from the SiN WG photonic mode for each mode, corresponding to the selected cross-sections.

Here, we explore the 1-ARM interferometer configuration, which offers a more compact footprint compared to the two-arm MZI configurations discussed above. In the 1-ARM architecture, switching is performed in the output photonic WG after the TO active element. This approach leverages constructive (maximum transmission) and destructive (minimum transmission) interference driven by the different plasmo-photonic modes supported by the TO WG stack. Specifically, the transition between constructive and destructive interference is achieved by utilizing the TO effect to induce the appropriate phase shift between the TO WG modes. Maximum total transmission is targeted at room temperature (i.e., $T_{max}={\left|t(T_{0})\right|}^{2}$), while minimum total transmission is obtained by applying a bias to the TO WG (i.e., $T_{min}={\left|t(T)\right|}^{2}$). To optimize the performance of the 1-ARM configuration, we explore various cross-sections by investigating a wide range of TO WG widths (Figure 11), as well as different low-index and high-index SiOC thicknesses (Figure 12). For each case, we aim to determine the optimal TO WG length combined with the applied bias that maximizes $T_{max}\;$ while keeping the minimum transmission within a tolerance of less than 1% (i.e., $T_{min}<0.01$).

In the 1-ARM interferometer optimization, we follow a similar approach with the MZI configurations. In Figure 11a, we show the $T_{max}$ of the device at its optimized length (as indicated in Figure 11b) as a function of the TO WG width and high-index SiOC thickness, with a fixed low-index SiOC thickness of 180 nm. Notably, in this configuration, we need to explore a higher range of high-index SiOC thicknesses, from 0.62 μm up to 1 μm, compared to the MZI configuration, which operates effectively within 0.3 μm to 0.6 μm. This adjustment is necessary to identify functional cross-sections with a minimum transmission below 1%. Through this exploration, we identify two regions of high maximum transmission (each with ~4 dB IL) that also achieve acceptable minimum transmission levels, corresponding to TO WG widths of 1.5 μm and 2.9 μm. We select the 1.5 μm width as the optimal choice since it requires only half the energy to achieve the desired temperature increase compared to the 2.9 μm width, due to its lower effective thermal capacity.

Subsequently, we investigate the impact of low-index and high-index SiOC thickness on the total transmission of the 1-ARM interferometer at the optimized width $W_{TO}=1.5\;\mu m$ (Figure 12a). We observe a clear trend toward a higher $T_{max}$ as the low-index SiOC thickness increases, similar to the behavior seen in the S-MZI architecture, albeit at thicker high-index SiOC layers. This difference can be attributed to the “strong photonic” regime observed in the S-MZI configuration ($t_{LI}=720\;nm$, $t_{HI}=160\;nm$), where efficient single-mode operation is achieved due to the coupling efficiency being concentrated in the fundamental photonic mode (Figure 8b(iii)). However, this cross-section is not suitable for 1-ARM operation since this architecture requires at least two modes to enable constructive or destructive interference at the end of the TO WG stack.

Therefore, we expand the phase space under investigation to include thicker high-index SiOC layers, ranging from 0.6 μm to 0.9 μm, to achieve multimode operation and identify the optimal cross-sections for 1-ARM functionality. In Figure 12b, we explore the optimized TO WG lengths corresponding to the total transmission results shown in Figure 12a. This analysis reveals regions of both long and short TO WG lengths, which are strongly influenced by the low- and high-index SiOC thicknesses. Focusing on a constant high-index SiOC thickness of $t_{HI}=660\;nm,$ we identify shorter optimized lengths, thereby ensuring low power consumption below 5 mW (as depicted in Figure 12c for four representative low-index SiOC thicknesses). Additionally, we present the total response of the 1-ARM interferometer as a function of normalized bias for the corresponding cross-sections (highlighted by the star points), showing a clear trend of increasing $T_{max}$, from 15% to 58%, as the low-index SiOC thickness increases (Figure 12d).

To understand this trend, we again plot the electric field spatial distribution (Re(Ey)) for the fundamental and three dominant plasmo-photonic modes supported by the TO WG for the representative cross-sections (Figure 12e). For the thin low-index SiOC, the electric field is strongly confined to the metal surface, with the coupling transmission shared among the different plasmo-photonic modes (Figure 12e(i,ii)). Similar to the MZI operation, we identify this region as “strongly plasmonic,” characterized by high propagation losses. The cross-section providing $T_{max}=34\%$ (4.7 dB IL) in the “strongly plasmonic” regime is found at $t_{LI}=300\;nm$, $t_{HI}=660nm$ with a TO WG length L=44.85 μm, and 2.6 mW power consumption (Figure 12e(ii)). As the low-index SiOC thickness increases, the two dominant modes with high coupling efficiency gradually shift from “strongly plasmonic” to “strongly photonic”, with the electric field decoupled from the metal and concentrated in the high-index SiOC and SiN WGs (Figure 12e(iii,iv)). In this regime, both propagation losses and coupling transmission to the other modes decrease, leading to an “almost” two-mode interference condition, which is desirable for efficient 1-ARM operation (Figure 12e(iv)). This cross-section provides $T_{max}=58\%$ (2.3 dB IL) in the “strongly photonic” regime, with $t_{LI}=900\;nm$, $t_{HI}=660\;nm,$ and a TO WG length L=75.93 μm at 4.1 mW power consumption. In summary, by tuning the low- and high-index SiOC thicknesses, one can exploit the best-in-class plasmo-photonic modes, transitioning from “strongly plasmonic” to “strongly photonic” operation, thus increasing $T_{max}$, albeit at the cost of higher power consumption.

## 4. Conclusions

In this work, we have theoretically designed and evaluated the performance of various TO interferometer architectures, including S-MZI, A-MZI, A-MZI-RR, and 1-ARM, for TM polarization at a 1550 nm telecom wavelength. These configurations were investigated within the framework of a CMOS-compatible design, utilizing a plasmonic Al/SiOC TO WG as the active switching element on a SiN photonic platform.

We developed a comprehensive opto-thermal model to optimize the performance of the different SiOC-based interferometers. This model serves as an efficient optimization tool for designing experiments, applicable not only to SiOC-based TO WGs but also to other similar devices. Specifically, we constructed a reliable semi-analytical model, which was implemented in an optimization process to determine the optimal device parameters for maximum transmission (minimum IL). In addition, we conducted thermal studies to analyze the structural parameters that ensure low power consumption, negligible thermal cross-talk between devices, and fast switching times. By utilizing the derived opto-thermal model, we extracted the optimal performance metrics for each configuration, including insertion losses, power consumption, and footprint (active length of the TO switcher).

**Figure 13:**
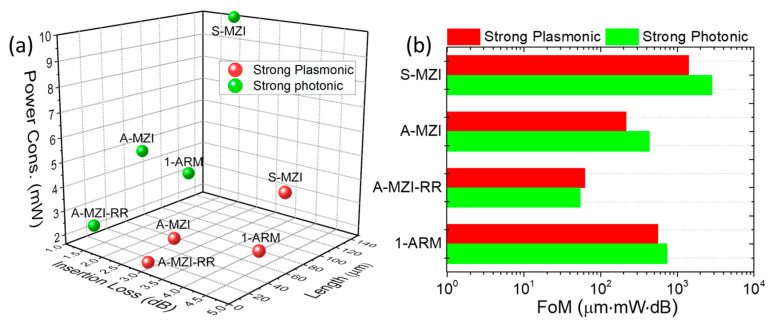
(**a**) Three-dimensional scatter plot of the optimized length, power consumption, and insertion losses for the different interferometer architectures. (**b**) Figure of merit for “strong plasmonic” and “strong photonic” operation for performance comparison between the interferometer architectures.

Our findings indicate that by tuning the geometric parameters of the plasmonic TO switching element (i.e., the low- and high-index SiOC thickness), one can exploit the best-in-class plasmo-photonic modes and transition from “strongly plasmonic” to “strongly photonic” operation. Each configuration presents specific advantages and disadvantages, which are summarized in Figure 13 for the different interferometer architectures.

Specifically, in Figure 13a, we summarize the optimized performance metrics for the different interferometer architectures under both “strong plasmonic” and “strong photonic” operations. By comparing the performance of each configuration at these two operational regimes, we observe that the “strong plasmonic” operation results in a shorter optimized length, leading to lower power consumption, but at the cost of higher insertion losses. In contrast, the “strong photonic” operation yields lower insertion losses but requires a longer optimized length and higher power consumption.

To evaluate and compare the performance of the explored architectures, we calculate the figure of merit (FOM) in units of μm⋅mW⋅dB for each type of operation and configuration. A lower FOM indicates higher efficiency, and we find that the A-MZI-RR configuration exhibits the best performance in this regard (Figure 13b). Specifically, the A-MZI-RR architecture demonstrates superior performance for both plasmonic and photonic operations, with minimal power consumption (1.6 mW for plasmonic, 2.1 mW for photonic), low insertion losses (2.8 dB for plasmonic, 1.1 dB for photonic), and a reduced length (14.4 μm for plasmonic, 22.7 μm for photonic).

In conclusion, our comparative analysis shows that SiN-based TO phase shifters achieve a lower FOM than alternative designs, as summarized in Table 1. This result underscores the strong potential of SiOC-based TO modulators in enabling energy-efficient, compact photonic integrated circuits (PICs) for advanced and emerging applications.

## Figures and Tables

**Table 1:** The performance metrics of existing TO switches operating at the 1550 nm telecom wavelength are compared to the results achieved in this work.

Performance Metrics of Switches *				
Switcher/ Parameters	Ins. Loss (IL) [dB]	Length (L) [μm]	Power Cons. (P) [mW]	FOM [dB⋅μm⋅mW]
2 × 2 MZI [24]	0.48	1000	64.4	30,912
4 × 4 switcher [25]	5.7	400	130	296,400
MZI tuners [31]	NA	270	350	NA
SiRN folder [32]	1.2	1200	8	11,520
DLSPP-MZI-PMMA [34]	24	46	16	17,664
DLSPP-MZI-CAP [35]	24	32.3	2.35	1822
DLSPP-AMZI-PMMA [37]	11	60	13.1	8646
DLSPP-AMZI-CAP [38]	10	40	12	4800
Plasmonic S-MZI [**]	4.9	62	4.7	1428
Plasmonic A-MZI [**]	3.1	28.5	2.5	221
Plasmonic A-MZI-RR [**]	2.8	14.4	1.6	65
Plasmonic 1-ARM [**]	4.7	44.9	2.6	549

* All the metrics are considered for a single switching operation. ** This work.

**Table 2:** Thermal properties of the materials used for thermal modeling.

Thermal Properties			
Material	K [W/mK]	Cp [J/KgK]	p [Kg/m^3^]
SiOC [48]	1.5	750	2300
SiN [49]	80	700	3170
SiO_2_ [50]	1.4	733	2200
Si [50]	149	712	2330

**Table 3:** Thermal model for the power consumption extraction at different TO WG widths and thermal configurations.

Thermal Properties				
Cases/Fit Parameters	*W*_TO_ = 2.1 μm Etched SiO_2_	*W*_TO_ = 2.1 μm Full SiO_2_	*W*_TO_ = 1.5 μm Etched SiO_2_	*W*_TO_ = 1.5 μm Full SiO_2_
a	3.91307 × 10^−4^	1.340 × 10^−3^	3.98041 × 10^−4^	1.770 × 10^−3^
b	8.56054	4.74353	9.95924	4.5718
c	2.84708	1.69812	3.46671	1.65783

**Table 4:** Pros and cons for the two operational regions of SiOC-based TO switcher.

Thin Low-Index SiOC	Thick Low-Index SiOC
strongly plasmonic	strongly photonic
high insertion losses	low insertion losses
shorter Lπ length	longer Lπ length
smaller footprint	larger footprint
lower consumption	higher consumption

## Data Availability

Data are contained within the article.
